# Commencements

**Published:** 1893-05

**Authors:** 


### COMMENCEMENTS.

### Baltimore College of Dental Surgery.

The fifty-third annual commencement exercises of the Baltimore College of Dental Surgery were held at Ford's Grand Opera House, on Monday afternoon, March 20, 1893, at two o'clock.

The annual oration was delivered by the Rev. H. Allen Tupper, Jr., D.D., and the valedictory by Robert F. Taylor, D.D.S.

The number of matriculates for the session was one hundred and thirty-one.

The degree of D.D.S. was conferred on the following graduates by Prof. R. B. Winder, M.D., D.D.S., Dean of the College:

| NAME | RESIDENCE | NAME | RESIDENCE |
|---|---|---|---|
| W. H. Gregg | Pennsylvania | Milton S. Merchant | Texas |
| Harry C. Griffith | Maryland | Francis H. Mulholland | Wisconsin |
| G. A. Hahn | Germany | Luther M. Parsons | Maryland |
| R. Edward Harbison | Pennsylvania | Erwin S. Rinehart | Texas |
| Chas. S Hoose | New York | Andrew W. Soule | Vermont |
| Claire G. Hamilton | Vermont | Frank W. Shegogue | Maryland |
| Albert C. Hays | New York | John W. Thompson | North Carolina |
| Fred Dwight Joy | New York | R. Franklin Taylor | Canada |
| Joseph Krainik | Roumania | B. F. Wendell | Pennsylvania |
| Charles A. Krantz | Maryland | John Wood | New York |

Total, 20

### Kansas City Dental College.

The eleventh annual commencement of the Kansas City Dental College was held at the Coates House, Kansas City, Mo., on Friday evening, March 3d, 1893.

The faculty address was delivered by Professor Charles H. Lester.

The number of matriculates for the session was seventy.

The degree of D.D.S. was conferred on the following graduates by C. B. Hewitt, D.D.S., President of the Faculty:

| NAME | RESIDENCE | NAME | RESIDENCE |
|---|---|---|---|
| Andrew W. Davis | Kansas | John H. Holke, M.D | Missouri |
| W. H. De Witt Dwight | Iowa | Richard J. Winn | Missouri |

Total, 4

### University of California—College of Dentistry.

The annual commencement exercises of the College of Dentistry of the University of California were held at Odd Fellows Hall, Thursday evening, March 9th, at eight o'clock.

The address on behalf of the Faculty was delivered by Harry P. Carlton, D.D.S., of the class of 1886.

The degree of D.D.S. was conferred on the following graduates by Martin Kellogg, A.M., President of the University:

| NAME | NAME |
|---|---|
| Edmund James Alger | William Andrew McQuitty |
| Reginald Heber Allen | George Hubbard Meredith |
| Jorge Arroyo | Edward Thomas Mervy |
| Frank Parker Ashworth | Edward Franklin Parr |
| William Nelson Avery | Corydon B. Root |
| Augustus Griffin Bennett, jr. | Ernest Frederick Schlott |
| Paul Stirling Coke | Frederick Schumacher |
| Emille William Davis | Walter Kendall Scott |
| Cecil Chalmers Dennis | James Graham Sharp |
| Albert Terrill Derby | Allen Holman Suggett, B. S. |
| William Caughlin Grove | Harry Francis Sullivan |
| Wilbur Hanford Halsey | Robert Lee Taylor |
| George Herman Huddy | William Emmett Taylor |
| Oscar Litchfield | Joseph Treleaven Thomas |

Total, 28

### University of Maryland—Department of Dental Surgery.

The annual commencement exercises of the Department of Dental Surgery of the University of Maryland were held at the Academy of Music, Baltimore, Thursday, March 16th, 1893.

The mandamus was read by the Dean, Prof. Ferdinand J. S. Gorgas, M.D., D.D.S.; the address to the graduates was delivered by the Rev. E. L. Watson; and the class oration by C. Howard Nicholson, Canada.

The number of matriculates for the session was one hundred and eight.

The degree of D.D.S. was conferred on the following graduates by Hon. S. Teackle Wallis, LL.D., Provost of the University.

| NAME | RESIDENCE | NAME | RESIDENCE |
|---|---|---|---|
| Fred Loveland Arnold | Rhode Island | William Lee Davis | California |
| S. DeLeon Avery | South Carolina | William W. Farmer | Virginia |
| William H. Barr | Canada | Roland E. Loucks | Canada |
| J. Edwin Boozer | South Carolina | Willie E. Minghini | West Virginia |
| Samuel A. Boyd | California | C. Howard Nicholson | Canada |
| Samuel M. Byers | Pennsylvania | John S. Rees | California |
| Norwood G. Carroll | North Carolina | Thomas R. Rowe | Rhode Island |
| Henry Winter Davis | Virginia | D. Fleming Sallis | Mississippi |

Total, 16

### Vanderbilt University—Dental Department.

The fourteenth annual commencement exercises of the Dental Department of this school were held in the University Chapel, Nashville, Tenn., on the evening of February 23d, 1893.

The number of matriculates for the session was one hundred and eleven.

The address to the class was deliverd by Prof. W. H. Morgan, M.D., D.D.S., Dean, and the class oration by Meander M. Bennett, D.D.S., of Tennessee.

The degree of D.D.S. was conferred by the Vice-Chancellor, W. F. Tillett, D.D., on the following persons:

| NAME | RESIDENCE | NAME | RESIDENCE |
|---|---|---|---|
| W. J. Baker | Alabama | Thos. S. Brown | Alabama |
| Jas. R. Beaucham | Mississippi | Meander M. Bennett | Tennessee |
| Wm. G. Downs | Indiana | John A. Johnson | Nova Scotia |
| John C. Minton | Texas | Le Roy Rentez | Pennsylvania |
| James D. Smith | Illinois | Geo. A. Hughes | Alabama |

Total, 10

### Philadelphia Dental College.

The thirtieth annual commencement of the Philadelphia Dental College was held in the College Ampitheatre, Philadelphia, Pa., on Wednesday, March 8, 1893, at 8. P. M.

The number of matriculates for the session was two hundred and eight.

The address to the graduates was delivered by Prof. H. I. Dorr, M.D., D.D.S., and the valedictory by Charles Bergstresser, D.D.S.

The degree of D.D.S. was conferred on the following persons:

| NAME | RESIDENCE | NAME | RESIDENCE |
|---|---|---|---|
| H. J. Bell | Illinois | W. H. Rothermel | Pennsylvania |
| Chas. Bergstresser | Kansas | F. H. Rowe | New Hampshire |
| W. E. Bowser | Pennsylvania | Chas. Ryckman | Pennsylvania |
| Wm. T. Butler | Pennsylvania | E. M. Sanderson | Florida |
| W. D. Campbell, M. D | Iowa | Edward Percy Saunders | Florida |
| E. Carty | New Jersey | Alfred Slocum | New York |
| H. C. Crease | Canada | Max R. Slough | Pennsylvania |
| George C. Davis, L. D. S | Canada | H. C. Sturdevant | Pennsylvania |
| W. H. Hisey | Ohio | John C. Sullivan | New York |
| J. S. C. Hovland | Norway | | |
| Louis N Lemieux | Canada | | |
| John B. Mahn | Pennsylvania | | |
| E. S. Messinger | Pennsylvania | | |
| J. T. Miller | Pennsylvania | | |
| H. B. Nase | Canada | | |
| C. W. Pitman | New Hampshire | | |

Total, 25

GRADUATED IN MAY, 1892.

| NAME | RESIDENCE |
|---|---|
| F. D. Crosby | Nova Scotia |
| Clifford B. High | Canada |
| Edgar R. Parker | Canada |
| Walter G. Van Horn | Pennsylvania |
| Walter L. Yerkes | New Jersey |

Total, 5

### Cincinnati College of Medicine and Surgery—Dental Department.

The second annual commencement of the Dental Department of the Cincinnati College of Medicine and Surgery was held at the Y. M. C. A. Hall, Cincinnati, O., on Tuesday evening, April 4th, 1893.

The opening address was delivered by Prof. J. R. Callahan, D.D.S., Dean of the Department, and the valedictory by Prof. E. W. Mitchell, M.D.

The number of matriculates for the session was thirty-nine.

The degree of D.D.S. was conferred on the following graduates by Prof. G. W. Harper, President of the Board of Trustees:

| NAME | NAME |
|---|---|
| Edwin J. French | Adolph Schramm |
| W. S. Martin | C. W. Simpkinson |

Total, 4

### Pennsylvania College of Dental Surgery.

The thirty-seventh annual commencement exercises of the Pennsylvania College of Dental Surgery were held at the Academy of Music, Philadelphia, Pa., on Tuesday, February 28th, 1893, at 12 M.

The annual address was delivered by Prof. Wilbur F. Litch, M.D., D.D.S.

The number of matriculates for the session was one hundred and ninety-seven.

The degree of D.D.S. was conferred on the following graduates by L. Minis Hays, M.D., President of the Board of Corporators:

| NAME | RESIDENCE | NAME | RESIDENCE |
|---|---|---|---|
| Lottie E. Benton | New York | Christopher Montag | Germany |
| Frank Brandreth | Pennsylvania | James Manning | Canada |
| Solomon Cox | Pennsylvania | Jeannette Oliver | New York |
| J. C. Condict | New Jersey | Thomas W. Powell | Pennsylvania |
| F. Pierce Farrow | New Jersey | A. M. Vernon Riggs | Pennsylvania |
| H. K. Frontz | Pennsylvania | Edwin Rogers | New York |
| A. A. Gribbin | New Jersey | John Richstoff | Sweden |
| Matilda Groth | Pennsylvania | Elton Stimmel | Pennsylvania |
| Henry W. Harrison | Kentucky | R. C. Talbot | Canada |
| John D. Hertz | Pennsylvania | H. H. Tory | Canada |
| Sophie E. Kuper | Germany | William B. Warren | New Jersey |
| Howard H. Laubach | Pennsylvania | | |

Total, 23

### New York College of Dental Surgery.

The twenty-seventh annual commencement exercises of the New York College of Dentistry were held in Chickering Hall, New York City, on Saturday evening, March 11, 1893.

The address to the graduates was delivered by J. Smith Dodge, M.D., and the valedictory by Ellison Hillyer, D.D.S.

The number of matriculates for the session was two hundred and eighty-six.

The degree of D.D.S., was conferred on the following persons by Wm. T. LaRoche, D.D.S., Vice-President of the Board of Trustees:

| NAME | NAME |
|---|---|
| Charles E. Aldous | George W. Mellor |
| Francis J. Agramonte | Louis Meriam |
| Henry R. Borst | James M. Nash |
| Fred. A. Bunting | Michael N. Nagle |
| Lester G. Brimmer | Philip W. Prior |
| Herman W. Botein | John O. Peterson |
| Edw. H. Berendsohn | Addison S. Peters |
| Ray P. Cummings | Harry L. B. Ryder |
| Robert S. Carman | Paul A. Reese |
| Jan. A. Chrzanowski | William H Russell |
| Octavio J. De Silva | William G. Sharp |
| Stanislaus A. Fischer | Louis F. W. Schrader |
| Charles P. Gates | Frederick L. Stanton |
| William M. Gould | Andrew J. Schuessler |
| Ellison Hillyer | Chas. L, Starbuck |
| William H. Hickok | Stephen V. Shea |
| Harry H. Hunt | Morris H. Smith |
| William F. Holmes | James O. Taylor, jr. |
| Richard E. Harvey | Dudley H. Tenney |
| Geo. A. Hammond | Howard M. Vere |
| Edward Hauck | George S. Wright |
| Thos. S. Jube, jr | Carl A. Williams |
| Thos. A. Kiernan | Frank W. Wickes |

Total, 46

### Northwestern University—Dental School.

The annual commencement exercises of the Dental School of the Northwestern University were held on Tuesday, April 25th, 1893, at Central Music Hall, Chicago, Ill.

The number of matriculates for the session was sixty-eight.

The degree of D.D.S. was conferred on the following graduates:

| NAME | NAME |
|---|---|
| Benjamen Merrill Ford | Murry Gordon Matteson |
| Jared Michael Gorman | Philip Albert Pyper |
| Charles Hazen Gale | Charles Arthur Templeton. |

Total, 6

### American College of Dental Surgery.

The seventh annual commencement exercises of the American College of Dental Surgery were held in the Grand Opera House, Chicago, Ill., on Tuesday, March 21, 1893, at 2:30 P. M.

The doctorate address was delivered by Rev. O. R. Gifford, and the valedictory by George T, Banzet, D.D.S.

The degree of D.D.S. was conferred on the following graduates by John S. Marshall, M.D., Dean of the Faculty:

| NAME | NAME |
|---|---|
| William E. Allen | Jane E. Kelley |
| Harry B. Adkins | Edmund W. Kester |
| George J. Brown | Adolphus E. McKenzie |
| Ernest Busch | Kauffman L. Meyers |
| George T. Banzet | John T. Murray |
| E. Lee Carter | Finley H. Mason |
| William J. Claassen | Theodore Menges |
| Bert C. Delano | Henry C. Miller |
| Fred C. Dorsch | Captain H. McComb |
| Charles H. Foster | Frederick E. Pilcher |
| Elmer E. Goudy | William B. Pearson |
| J. F. Goodrich | M. Antoine Sayler |
| Mary C. Harter | Julius C. Schneider |
| John Harler | Karl W. Woodward |

Total, 28

### Ohio College of Dental Surgery.

The forty-seventh annual commencement of the Ohio College of Dental Surgery was held at the Young Men's Christian Association Hall, Cincinnati, on Wednesday evening, March 15th, 1893, at 8 o'clock.

The number of matriculates for the session was one hundred and twenty-one.

The annual address was delivered by the Rev. Dudley Rhodes, and the class oration by Charles Leslie Casey, of Cambridge, Ohio.

The degree of D.D.S. was conferred on the following persons by D. W. Clancey, M.D., D.D.S., Vice-President of the Board of Trustees:

| NAME | NAME |
|---|---|
| Pearl Wilbur Applegate | Max Julius Hans Martin |
| Jesse Sydenham Bailey | Philip Martin Offutt |
| Sharon King Bailey | U. Clarence Purdum |
| Charles Leslie Casey | David Este Sheehan, jr. |
| Richard Ambrasa Foley | Victor Trager |
| John Baptist Hayes | Julius Calvin Van Kirk |
| John Sheperd Hussey | Howard Austin Whiteside |
| Francis Allen Lush | Total, 15 |

### Southern Medical College—Dental Department.

The sixth annual commencement exercises of the Dental Department of the Southern Medical College were held at De Give's Opera House, Atlanta, Ga., March 2d, 1893, at 8 o'clock P. M.

The number of matriculates for the session was one hundred.

The annual address was delivered by T. S. Powell, M.D., and the valedictory by R. A. Caswell, D.D.S., of Florida.

The degree of D.D.S. was conferred by the President, T. S. Powell, M.D., upon the following persons:

| NAME | RESIDENCE | NAME | RESIDENCE |
|---|---|---|---|
| C. J. Alvis | Alabama | E. C. Smith | South Carolina |
| H. E. Carpenter | Georgia | William R. Tyler | Georgia |
| R. A. Casswell | Florida | W. H. Treadwell | Georgia |
| I. L. Dedge | Georgia | C. G. White | South Carolina |

Total, 8

### Royal College of Dental Surgeons of Ontario.

The number of matriculates for the session at the Royal College of Dental Surgeons of Ontario, Toronto, Canada, was ninety-one.

At the annual meeting of the Board of Directors, held March 30th, 1893, certificates of license to practice dentistry and the title of L.D.S. were granted to the following persons, who had fully complied with the curriculum and passed the examinations:

| NAME | RESIDENCE | NAME | RESIDENCE |
|---|---|---|---|
| W. W. Alton, D.D.S | Hamilton | J. W. Marshall | Shelburne |
| Joseph Brooks, D.D.S | Alliston | W. M. McGuire | Waterford |
| Geo. Albert Bentley | Forest | W. T. McGorman | St. Marys |
| Fred. T. Coghlan | Guelph | E. A. Peaker | Toronto |
| Harold Clark | Toronto | J. C. S. Robertson | Ottawa |
| J. G. Coram | Drayton | R. J. Robins | Warminster |
| W. A. Crowe, D.D.S | Tara | D. E. Russell | Hamilton |
| S. R. Clemes | Thornbury | C. J. Rodgers | Toronto |
| D. I. Dulmadge | Brighton | Colon E. Smith | Bothwell |
| Horace E. Eaton, D.D.S | Toronto | Milo H. Steele, D.D.S | Arnprior |
| George S. Fowler | Palmerston | J. A. Sanders | Kemptville |
| Edwin Forster | Toronto | Geo. D. Scott | Port Hope |
| E. S. Hardie | Tilsonburg | Charles Thompson | Hamilton |
| E. A. Harrington | Toronto | J. M. Turnbull | Owen Sound |
| George Hicks | Talbotville | Nelson Wager, B. A. | Napanee |
| John Irwin | Collingwood | C. H. Wortman | Napanee |
| R. J. Lougheed | Toronto | John E. Wilkinson | Brampton |
| James Loftus | Toronto | C. H. Waldron, B. A. | Toronto |
| Robert Meek | Toronto | | |

Total, 37

### The Western Dental College of Kansas City.

The third annual commencement of the Western Dental College was held at the Grand Avenue M. E. Church, Kansas City, Mo., on Tuesday evening, March 7th, 1893.

The annual address was delivered by the Rev J. Z. Armstrong, Ph.D., LL.D.; and the valedictory by Dr. Clifton Covert.

The number of matriculates for the session was ninety-three.

The degree of D.D.S. was conferred on the following persons by the Dean, D. J. McMillen, D.D.S.:

| NAME | NAME |
|---|---|
| W. W. Bryan | I. L. Collison |
| A. E. Bonnell | A. J. Raffington |
| C. C. Covert | W. J. Walkins |

Total, 6

### Alabama College of Dental Surgery.

The first annual commencement exercises of the Alabama College of Dental Surgery were held at the Aldhous Building, Bridgeport, Ala., February 24th, 1893.

The number of matriculates for the session was fifteen.

Addresses were delivered by Rev. W. A. C[illegible]ow, T. M. Allen D.D.S., W. K. Spiller and Charles A. Holmes, President of the Board; and the valedictory by A. Irene Yokum.

The degree of D.D.S. was conferred on the following persons, who had completed the third term, by Charles A. Holmes, President of the Board of Trustees:

| NAME | NAME |
|---|---|
| Sandford W. Allen | A. Irene Yokum |
| Henry Clay Stephens | |

Total, 3

### Meharry Medical College—Dental Department.

The seventh annual commencement of the Meharry Dental Department of Central Tennessee College was held in connection with that of the Medical and Pharmaceutical Departments, at the Gospel Tabernacle, Nashville, Tenn., on Tuesday evening, February 7th, 1893.

The address to the graduating class was delivered by Dr. J.

L. M. Curry, D.D., and the valedictory by Alonzo M. Wilkins, of Georgia.

During the past session seven students have been enrolled in the Dental Department.

Rev. J. Braden, President of the College, conferred the degree of Doctor of Dental Surgery on S. Richard Thompson, of Tennessee, and Alonzo M. Wilkins, of Georgia.

